# Integration of CD45-positive leukocytes into newly forming lymphatics of adult mice

**DOI:** 10.1007/s00418-015-1399-y

**Published:** 2016-01-09

**Authors:** K. Buttler, M. Lohrberg, G. Gross, H. A. Weich, J. Wilting

**Affiliations:** Department of Anatomy and Cell Biology, University Medical School Göttingen, Göttingen, Germany; Department of Gene Regulation, Helmholtz Centre for Infection Research, Brunswick, Germany; Department of Chemical Biology, Helmholtz Centre for Infection Research, Brunswick, Germany

**Keywords:** Lymphatic endothelial cell, Lymphangioblast, Lymphangiogenesis, Lymphvasculogenesis, Endothelial progenitor cell, Mesenchymal stem cell

## Abstract

The embryonic origin of lymphatic endothelial cells (LECs) has been a matter of controversy since more than a century. However, recent studies in mice have supported the concept that embryonic lymphangiogenesis is a complex process consisting of growth of lymphatics from specific venous segments as well as the integration of lymphangioblasts into the lymphatic networks. Similarly, the mechanisms of adult lymphangiogenesis are poorly understood and have rarely been studied. We have recently shown that endothelial progenitor cells isolated from the lung of adult mice have the capacity to form both blood vessels and lymphatics when grafted with Matrigel plugs into the skin of syngeneic mice. Here, we followed up on these experiments and studied the behavior of host leukocytes during lymphangiogenesis in the Matrigel plugs. We observed a striking co-localization of CD45^+^ leukocytes with the developing lymphatics. Numerous CD45^+^ cells expressed the LEC marker podoplanin and were obviously integrated into the lining of lymphatic capillaries. This indicates that, similar to inflammation-induced lymphangiogenesis in man, circulating CD45^+^ cells of adult mice are capable of initiating lymphangiogenesis and of adopting a lymphvasculogenic cellular differentiation program. The data are discussed in the context of embryonic and inflammation-induced lymphangiogenesis.

**Electronic supplementary material:**

The online version of this article (doi:10.1007/s00418-015-1399-y) contains supplementary material, which is available to authorized users.

## Introduction

The embryonic origin of the lymphatic vascular system has been studied for more than a hundred years, but controversies still exist. Florence Sabin was among the first to study embryonic lymphangiogenesis, and stated that, in pigs, lymphatic vessel formation takes place via sprouting from specific segments of deep embryonic veins (Sabin 1909). In contrast, Kampmeier postulated an additional mesenchymal origin of lymphatics (Kampmeier 1912). Our insight into the mechanisms regulating lymphatic vessel formation has greatly increased by the imaging of labeled cell lines in living animals, predominantly supporting Kampmeier’s hypothesis. For instance, in zebrafish embryos, precursor cells were found to migrate from the cardinal vein to the horizontal myoseptum to generate parachordal lymphangioblasts in this area (Küchler et al. 2006; Yaniv et al. 2006; Hogan et al. 2009; Isogai et al. 2009). These parachordal lymphangioblasts could be pursued to migrate along arteries and to remodel into major trunk lymphatic vessels thus forming the thoracic duct (Yaniv et al. 2006; Bussmann et al. 2010; Cha et al. 2012). The data provided evidence for an intermediate mesenchymal phase of lymphangiogenesis in fish. Although a strong conservation of genes controlling lymphangiogenesis could be detected between zebrafish and mammals (Schulte-Merker et al. 2011; Koltowska et al. 2013), in higher vertebrates the lymphatic vascular system is more complex than in fish, which hardly possess any epifascial, dermal lymphatics. Moreover, organ-specific lymphatic vascular patterns can be seen in higher vertebrates, but the cellular origins of embryonic lymphatics in diverse compartments of the body are still subject of controversial discussions.

Apart from the embryo, lymphangiogenesis takes place under pathological conditions (Wilting et al. 2009; Tammela and Alitalo 2010). In murine experimental models employing the lung and the skin, it could be shown that acute inflammation is able to induce lymphangiogenesis effectively (Pullinger and Florey 1937; Baluk et al. 2005). Thereby, pro-inflammatory factors such as interleukin-1 and tumor necrosis factor-*α* induce the up-regulation of the lymphangiogenic vascular endothelial growth factor-C (VEGF-C; Cha et al. 2007). But also in man, acute inflammation is a strong pro-lymphangiogenic stimulus (Kerjaschki et al. 2006), whereas chronic inflammation obviously has a deleterious effect on the lymphatics. After repeated inflammation collectors seem to loose their contractility, lymph then coagulates and the vessels subsequently obliterate (Földi and Földi 2003). During inflammation-induced kidney transplant rejection in man, massive lymphangiogenesis into the parenchyma of the rejected kidneys has been observed; additionally, there was evidence for the integration of circulating cells into the lining of the newly developing lymphatics (Kerjaschki et al. 2006). However, it is still a matter of debate, if the mechanisms of lymphangiogenesis in the mouse recapitulate those in man.

We have recently shown that endothelial progenitor cells (EPCs) isolated from the lungs of adult mice have the capacity to form both blood vessels and lymphatics, when grafted into the skin of mice using Matrigel^®^ plugs. Thereby, hem- and lymphangiogenesis were initiated via stimulation of the EPCs either by direct application of growth factors such as VEGF-A and basic fibroblast growth factor (FGF; Schniedermann et al. 2010), or by co-injection of mesenchymal stem cells (MSCs), which secrete a variety of angiogenic factors. Thereby, we observed no participation of MSCs in the formation of the vascular wall (Buttler et al. 2014). Here, we followed up on these experiments and studied the behavior of host leukocytes during lymphangiogenesis in the Matrigel^®^ plugs. We found a significant co-localization of CD45^+^ leukocytes with the developing lymphatics, and a considerable number of these cells were obviously integrated into the lining of newly formed lymphatics. This led us to the conclusion that, similar to the mechanisms of inflammation-induced lymphangiogenesis in man, circulating CD45^+^ cells of adult mice are capable of initiating a lymphvasculogenic program.

## Materials and methods

### Animals

Lung-derived EPCs and bone marrow-derived mesenchymal stem cells (MSCs) were isolated from C57/Bl.6 mice, and the cells were grafted into adult C57/Bl.6 mice. For the transplantation experiments, we used 8–12 weeks-old female mice. All experiments were approved by our local institutional animal care committee and the Lower Saxony state council on animal care (LAVES). The experiments corresponded to the requirements of the American Physiological Society.

### Isolation and culture of EPCs and MSCs

All cells were isolated from C57/Bl.6 mice. EPCs were isolated from mouse lungs using a magnetic cell separation method that has already been described by Schniedermann et al. (2010). Briefly, the lungs of adult mice were dissected after perfusion, minced and digested using collagenase A. A single cell suspension of the collected cells was produced with a 40 μm cell strainer, and CD31^+^ cells were cultivated after magnetic activated cell sorting using anti-CD31-coated Dynabeads^®^ (Thermo Fischer Scientific). After 8–10 days, cells were separated again using FACS sorting with anti-CD31 antibodies.

MSCs were isolated from bone marrow as described by Soleimani and Nadri (2009). Shortly, mice were killed by cervical dislocation and the hind limbs were separated from the trunk after removal of the skin. Muscles and connective tissue was then removed from the tibia and the femur under sterile conditions. To harvest bone marrow, the distal ends of tibia and femur were cut off and bone marrow was flushed out with PBS by insertion of a needle into the cancellous bone. The cell suspension was then filtered, and bone marrow cells were transferred into culture dishes. The cells were used between passages 6–10 (MSCs) and 8–24 (EPCs). As in our previous studies (Schniedermann et al. 2010; Hoffmann et al. 2006), MSCs and EPCs were cultured in DMEM enriched with FCS in 24- and 6-well plates (Nunc^®^). EPCs were cultured in gelatine-coated wells or cell culture flasks.

### Assessment of vessel formation in vivo

For the in vivo experiments, MSCs and EPCs were trypsinized and counted. For the experiments with each single cell type, 1 × 10^6^ cells were used. When the two cell types were combined in a 1:1 mixture, 0.5–1 × 10^6^ cells were used for each cell type. Cells were centrifuged and the pellets were dissolved in 300 µl of cold Matrigel^®^ (Corning, Wiesbaden, Germany). The mice were shaved and mildly sedated by intraperitoneal injection of ketamine (100 mg/kg body weight). Matrigel plugs with or without cells were injected subcutaneously into the left and right dorsal lumbar region (two injections per animal). After 7–9 days, the animals were killed, the skin was dissected, the Matrigel plugs photographed in situ, excised and fixed with 4 % paraformaldehyde (PFA). The specimens were rinsed in PBS, immersed with saccharose and embedded in tissue freeze medium (Neg-50, Richard-Allan Scientific, Mi). The numbers of experiments were: Matrigel without cells (*n* = 11), Matrigel with EPCs (5), Matrigel with MSCs (6), Matrigel with EPCs and MSCs (26).

### Histology and immunohistology

Frozen specimens were sectioned at 16 µm, mounted on slides, and stained with hematoxylin and eosin (HE). For the immunofluorescence studies, non-specific binding was blocked by the incubation with 2 % bovine serum albumin (BSA) for 1 h prior to the incubation with the primary antibodies. Primary antibodies were anti-mouse CD31 (rat clone MEC13.3; 1:50, BD), anti-podoplanin (syrian hamster clone 8.1.1, 1:1000, Hybridoma Bank, Iowa), anti-Prox1 (rabbit polyclonal, 1:500, Reliatech, Germany), and anti-mouse CD45 (rat monoclonal, 1:50, BD). After incubation with the primary antibodies for 1 h, sections were rinsed and the secondary antibodies were applied: goat anti-rat Alexa594/or Alexa488, donkey anti-rabbit Alexa488, goat anti-hamster Alexa594/or Alexa488 (all from Invitrogen). Dapi was used to counter-stain all nuclei. The sections were mounted under coverslips using Fluoromount-G (Southern Biotechnology, US) and studied with Axio Imager Z1 with ApoTome device (Zeiss, Göttingen, Germany).

## Results

**Fig. 1:**
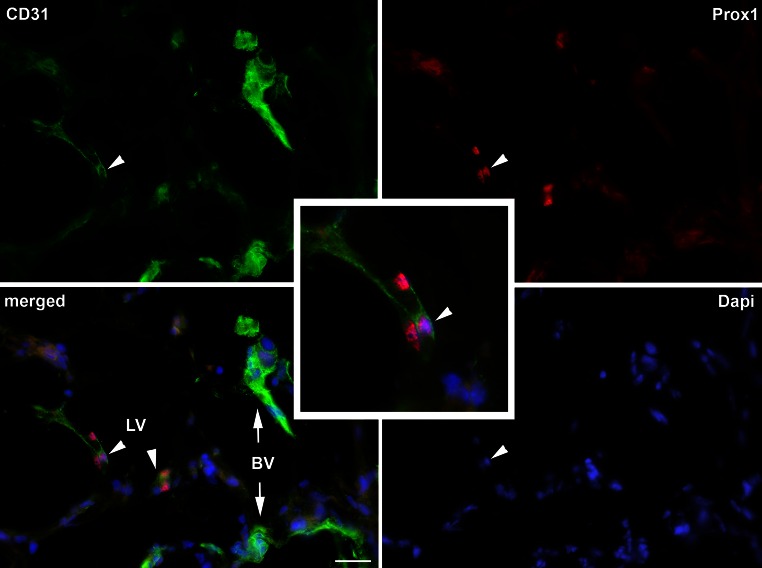
Verification of lymphatics in the Matrigel plugs with *CD31* (*green*) and *Prox1* (*red*) antibodies. The pan-endothelial marker *CD31* stains blood vessels (*BV*) strongly and lymphatics (*LV*) weakly. *Prox1* marks the nuclei of LECs (*arrowheads*) as shown at higher magnification in the *inset*. Nuclei are counter-stained with Dapi (*blue*). *Bar* 30 µm

**Fig. 2:**
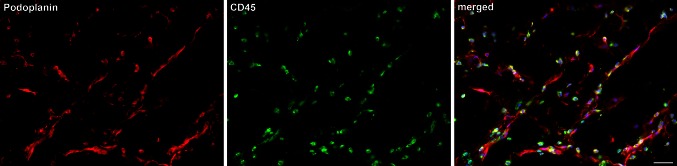
Lymphatics in the Matrigel plugs are closely associated with leukocytes. Networks of lymphatic capillaries are demonstrated with the LEC marker podoplanin (*red*). Note that the distribution of leukocytes, stained with anti-CD45 antibodies (*green*), closely follows the pattern of the lymphatics. Nuclei are counter-stained with Dapi (*blue*). *Bar* 50 µm

**Fig. 3:**
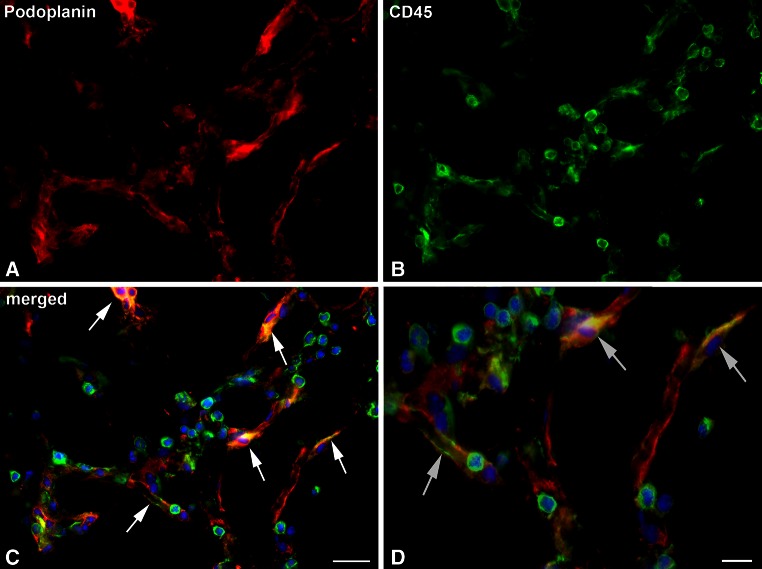
A subpopulation of cells in the lymphatic networks are positive for both podoplanin and CD45. **a** *Podoplanin* (*red*). **b** *CD45* (*green*). **c** *Merged* picture. Besides round CD45-positive cells, there are endothelial-like cells that co-express podoplanin and CD45 (*arrows*). Nuclei are counter-stained with Dapi (*blue*). *Bar* 25 µm. (Modified from: Buttler et al. 2014; Springer license no.: 3699330937457). **d** Higher magnification of (**c**) note endothelial-like cells that co-express podoplanin and CD45 (*arrows*), indicating integration of leukocytes into developing lymphatic networks. *Bar* 10 µm

**Fig. 4:**
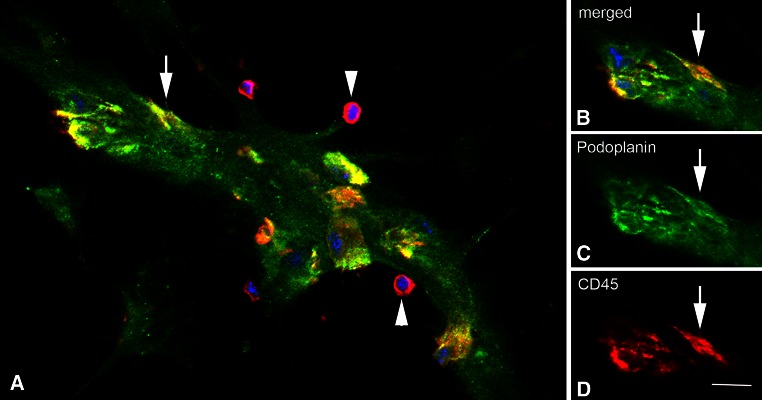
Endothelial-like cells in the lymphatic sinusoids are positive for both podoplanin and CD45. **a** *Merged* picture of a *podoplanin* ^+^ (*green*) lymphatic sinus. *CD45* is shown in *red*. Besides *round* CD45^+^ leukocytes (*arrowheads*), there are endothelial-like cells (*yellow*) that co-express the two markers. **b**–**d** Merged picture and separate channels showing the cell marked with an *arrow* in (**a**). *Bar* 10 µm

**Fig. 5:**
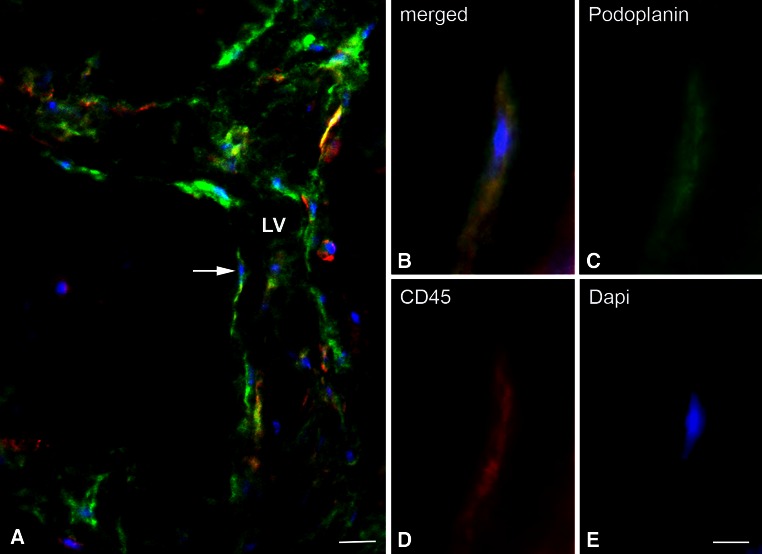
Cells lining newly formed lymphatics express podoplanin and CD45. **a** Merged picture of a lymphatic vessel (*LV*) in the Matrigel plug, stained for *podoplanin* (*green*) and *CD45* (*red*). Nuclei are counter-stained with *Dapi* (*blue*). The vessels are lumenized and have two branches to the *left*. *Bar* 20 µm. **b**–**e** *Merged* picture and separate channels showing the cell marked with an *arrow* in (**a**). The cell lining the vessels expresses both *podoplanin* and *CD45*. *Bar* 8 µm

In all experiments performed with Matrigel^®^ plugs containing a 1:1 mixture of EPCs and MSCs, the stereomicroscopic inspection revealed the presence of blood vessels in the plugs already after 7–9 days. These vessels were obviously highly fragile since careful dissection of the skin always resulted in hemorrhage in the plug region. Matrigel^®^ controls and plugs containing only each single cell type were macroscopically and microscopically free of vessels, as it was shown in detail recently (Buttler et al. 2014). The development of vessels in the EPC/MSC-containing plugs was verified by immunofluorescence using antibodies against CD31/PECAM1. In normal tissues, blood vascular endothelial cells (BECs) are strongly CD31/PECAM1-positive, whereas lymphatic endothelial cells (LECs) are just weakly positive and characterized by their nuclear expression of the Prox1 transcription factor (Wilting et al. 2002). Both types of expression pattern could be found in EPC/MSC-containing Matrigel^®^ plugs, clearly documenting the development of blood vessels and lymphatic networks (Fig. 1). The newly formed vessels were made up of a mosaic containing grafted EPCs and host endothelial cells. We did not find any signs for the integration of MSC into these vessels (Buttler et al. 2014). Studies using antibodies against the pan-leukocyte marker CD45 revealed massive infiltration of leukocytes into the experimental plugs, indicating an inflammatory-type of lymphangiogenesis, although a syngeneic model was used in our grafting experiment. We observed a significant co-localization of host leukocytes with the podoplanin–positive lymphatic capillary networks in the plugs (Fig. 2), indicating an active participation of the leukocytes in adult lymphangiogenesis. Interestingly, a subpopulation of CD45^+^ cells assumed endothelial-like morphology and was obviously integrated into the newly forming lymphatic networks (Fig. 3). In many cases, like those shown in Figs. 2 and 3, the new lymphatics formed delicate capillary-like networks, but we also observed sinusoid-like lymphatics. Again, round CD45^+^ leukocytes could clearly be distinguished from endothelial-like cells, which co-expressed CD45 and podoplanin (Fig. 4). In rare case we found lymphatics with a wide lumen. In such cases we observed cells that obviously lined the lumen of the vessel (Fig. 5; Suppl. Movie 1). Our data provide strong evidence for the existence of leukocytes with lymphvasculogenic potential in adult mice.

## Discussion

### Lymphangiogenic potential of endothelial progenitor cells

We have previously shown that the lung of mice contains EPCs that possess the potential to differentiate into both BECs and LECs (Schniedermann et al. 2010; Buttler et al. 2014). Thereby, the EPCs exert their hem- and lymphangiogenic program only after exogenous stimulation, either by direct application with VEGF-A and FGF, or after co-transplantation with MSCs (Melero-Martin et al. 2008; Schniedermann et al. 2010; Lin et al. 2012; Buttler et al. 2014). It has to be pointed out that rodents have the capacity to regenerate lung tissue after resection, which has not been observed in the human. Regeneration of mouse lung is accompanied by the formation of new blood vessels (Konerding et al. 2012); however, the development of lymphatics has not been studied in this model. In our experiments, the lymphatics in the Matrigel plugs are formed by a mosaic of grafted EPCs and host-derived LECs, which was shown by the use of GFP-labeled EPCs (Buttler et al. 2014). Since we used a syngeneic mouse model, it is admissible to speculate that autologous patient-derived EPCs may behave in a similar way and integrate into newly developing lymphatic networks, which might provide a therapeutic option for the treatment of lymphedema.

### Leukocytes in murine lymphangiogenesis

Here, we studied lymphangiogenesis in adult mice and observed a striking co-localization of new lymphatics with CD45^+^ leukocytes. In an embryonic mouse model of lymphangiogenesis, a population of myeloid cells, characterized by the expression of the tyrosine kinase Syk, was found to express the lymphangiogenic factors VEGF-C and -D, as well as various chemokines. The Syk^+^ cell population comprises M2-polarized mononuclear cells and regulates developmental lymphangiogenesis (Böhmer et al. 2010). The effect of the Syk^+^ cells could be inhibited in vivo by the application of soluble VEGFR-3, a specific inhibitor for VEGF-C and -D (Böhmer et al. 2010). An important function for VEGF-C-secreting macrophages and neutrophils has been reported in various murine lymphangiogenesis models (Schoppmann et al. 2002; Baluk et al. 2005; Gordon et al. 2010).

### Inflammation-induced lymphangiogenesis

Our studies revealed an obvious co-localization of CD45^+^ leukocytes with the newly forming lymphatic networks. The accumulation of leukocytes is known as a characteristic feature of inflammation. Consistent with this, EPCs and MSCs secrete chemoattractants such as CXCL1, 10 and 16, as well as MCP1 (Buttler et al. 2014). Among others, these factors attract macrophages, which can release angiogenic proteins such as VEGF-A and VEGF-C (Schoppmann et al. 2002; Mantovani et al. 2006). The contribution of macrophages to inflammation-induced lymphangiogenesis could, among others, be demonstrated in a macrophage-deficient mouse model (Kubota et al. 2009). Since we did neither observe any significant numbers of leukocytes in the Matrigel controls, nor in the experiments with EPCs, it is obvious that the attraction of leukocytes is induced by the grafted MSCs. As we have used a syngeneic C57/Bl.6 mouse model, it is unlikely that leukocyte immigration is a sign for host-versus-graft reactions.

Human cornea and kidney transplant rejection shows most clearly that inflammation is a potent inducer of lymphangiogenesis. In the cornea, inflammation induces growth of lymphatics, which remain clinically invisible. Lymphangiogenesis then facilitates trafficking of antigen-presenting cells from the cornea to draining lymph nodes, induction of hypersensitivity and corneal rejection (Chen et al. 2007). Similarly, in renal transplant rejection, inflammation is associated with a 50-fold increase in lymphatic vessel density and invasion of lymphatics into the tubulointerstitial stroma (Kerjaschki et al. 2004). Thereby, host cells, which obviously represent circulating lymphendothelial progenitor cells, integrate into the endothelial lining and contribute to the newly developing lymphatic networks (Kerjaschki et al. 2006). In this respect, the mouse model presented in our study deeply reflects the mechanisms of human inflammation-induced lymphangiogenesis.

### Embryonic lymphangiogenesis

Regarding the embryonic development of the lymphovascular system, it is generally accepted that lymph sacs, the first histologically detectable structures of the lymphatic system, are formed by outgrowth of pre-lymphatic clusters from the cardinal veins (Sabin 1909; Pollmann et al. 2014). In recent years, the lymph sacs were proposed to represent the sole origin of the whole lymphatic system of mice (Srinivasan et al. 2007). However, an opposing theory claiming the existence of mesenchymal precursor cells, so-called lymphangioblasts, as a second source for the lymphatic vasculature is getting more and more conceivable. In 1910, Huntington and McClure were the first to speculate on the existence of mesenchymal precursor cells for LECs (Huntington and McClure 1910). Further evidence was found by analyzing the expression patterns of the LEC marker Prox1 in mesenchymal cells of *Xenopus*, chicken and mouse embryos, revealing scattered cells of lymphatic character supposed to represent lymphangioblasts (Schneider et al. 1999; Ny et al. 2005; Buttler et al. 2006). Evidence for lymphangioblasts in both the intra-embryonic and the extra-embryonic mesoderm of avian embryos was provided with quail-chick-transplantation experiments. Mesodermal cells of 2- to 3-day-old quail embryos are able to integrate into the host’s lymph sacs, lymph hearts and allantoic lymphatics when transplanted into corresponding regions of chick embryos (Papoutsi et al. 2001; Wilting et al. 2000, 2006; Valasek et al. 2007). As cells of the macrophage/monocyte lineage were found to contribute to lymphangiogenesis in inflammation, they were suggested to participate in the embryonic formation of the lymphatic system, too. Trans-differentiation of macrophages to LECs was suggested, but, although Lyve-1-positive macrophages were found capable of integrating into the wall of developing lymphatics, Prox1 expression was not detected. Hereby, trans-differentiation could be excluded (Gordon et al. 2010).

In mammals, the formation of embryonic blood vessels in a bipartite way is generally accepted, meaning there is (1) de novo formation of vessels from angioblasts (vasculogenesis) and (2) growth of vessels from preexisting ones by sprouting, splitting and intercalation (angiogenesis; Risau 1997). The existence of lymphangioblasts in mammals has been negated by a number of researchers in the field. However, recently elegant studies on mice have shown that a significant part of the dermal lymphatic vasculature is formed independently of venous sprouts. Using lineage-tracing experiments they could depict a non-venous origin of LECs in the lumbar skin and along the dorsal midline. In a process referred to as lymphvasculogenesis, these cells were found to assemble into clusters, subsequently generating vessels (Martinez-Corral et al. 2015). In a second study the same group could show that parts of the mesenteric lymphatic vasculature are formed from progenitors of hemogenic endothelial origin, thus representing further evidence for the lymphatic system being of multiple origin (Stanczuk et al. 2015). The hemogenic endothelium is the intra-embryonic source of blood cells. Together, the data show that murine lymphatics are derived from venous and non-venous origin, and the development of blood cells and LECs is closely connected. As shown in our study, the mechanisms of adult lymphangiogenesis recapitulate to a great extent those of the embryo. Lymphvasculogenic and lymphangiogenic mechanisms exist side by side. Thereby, adult lymphangioblasts like their embryonic counterparts may also be derived from hemogenic precursor cells.

## Electronic supplementary material

Below is the link to the electronic supplementary material.

Suppl. Movie 1 Movie of the specimen shown in Fig. 5. Note that numerous CD45^+^ cells (red) are found in the wall of the podoplanin^+^ (green) lymphatic vessel (MOV 8732 kb)
